# Supplementary material for: Comparing the Effects of Intracellular and Extracellular Magnetic Hyperthermia on the Viability of BxPC-3 Cells
Source: Nanomaterials (Basel). 2020 Mar 24;10(3):593. doi: 10.3390/nano10030593 (PMC7153512; doi:10.3390/nano10030593)
Supplement: Supplementary file 1 [file nanomaterials-10-00593-s001.pdf]

Supplementary

Comparing the Effects of Intracellular and Extracellular Magnetic Hyperthermia on the Viability of BxPC-3 Cells

Gary Hannon [1], Anna Bogdanska [1], Yuri Volkov [1,2,3,4,*], and Adriele Prina-Mello [1,2,3,*]

1 Nanomedicine and Molecular Imaging Group, Trinity Translational Medicine Institute, Dublin 8, Ireland; hannonga@tcd.ie (G.H.); abogdans@tcd.ie (A.B.)
2 Laboratory of Biological Characterization of Advanced Materials (LBCAM), Trinity Translational Medicine Institute, Trinity College Dublin, Dublin 8, Ireland
3 Advanced Materials and Bioengineering Research (AMBER) centre, CRANN institute, Trinity College Dublin, Dublin 2, Ireland
4 Department of Histology, Cytology and Embryology, First Moscow State Sechenov Medical University, Moscow 119992, Russia
* Correspondence: YVOLKOV@tcd.ie (Y.V.); PRINAMEA@tcd.ie (A.P.M.) Tel.: +353-1896-3259 (A.P.M.)

Received: 6 February 2020; Accepted: 22 March 2020; Published: date

A.

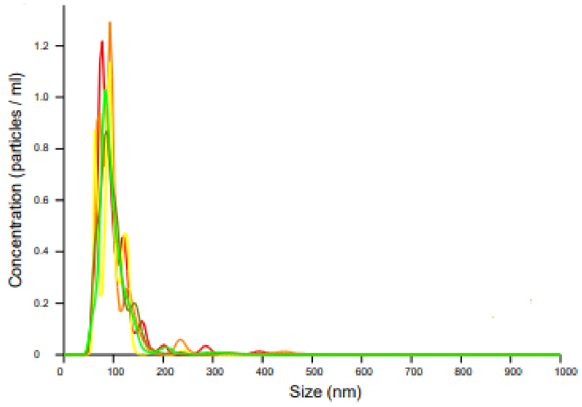

Concentration (particles / ml)
1.2
1.0
0.8
0.6
0.4
0.2
0
0
100
200
300
400
500
600
700
800
900
1000
Size (nm)

B.

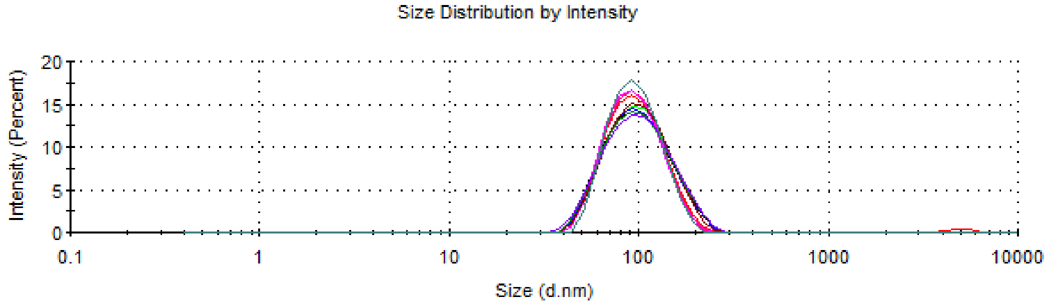

Size Distribution by Intensity
Intensity (Percent)
20
15
10
5
0
0.1
1
10
100
1000
10000
Size (d.nm)

C.

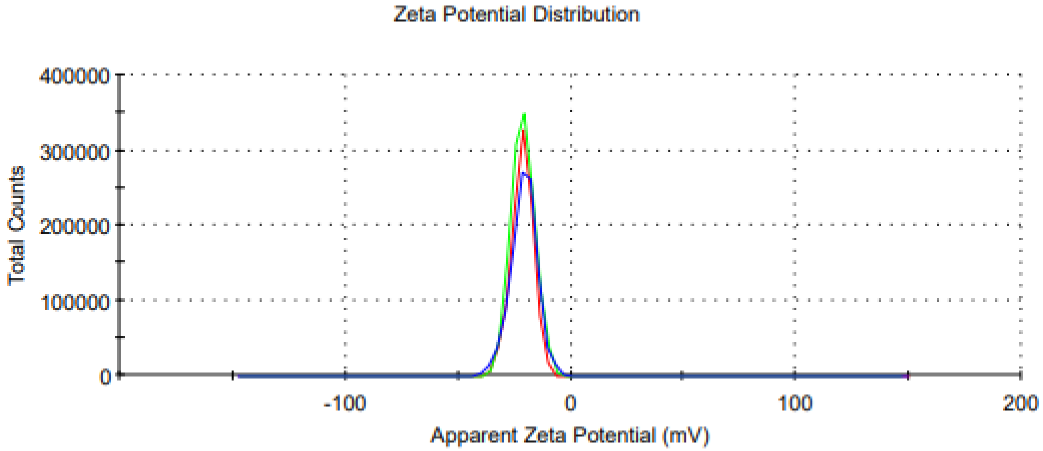

Zeta Potential Distribution
Total Counts
400000
300000
200000
100000
0
-100
0
100
200
Apparent Zeta Potential (mV)

D.

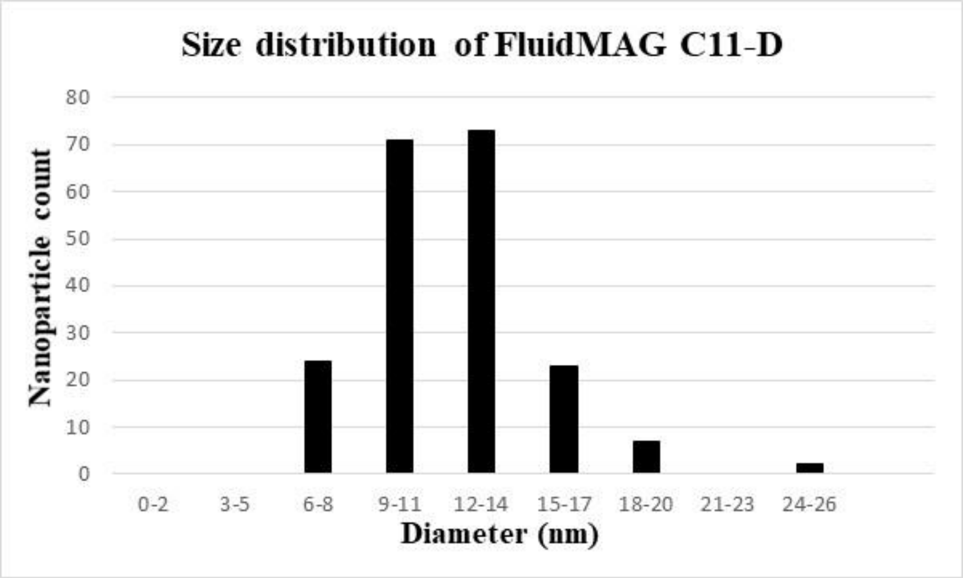

Figure S1: Nanoparticle tracking analysis, dynamic light scattering and transmission electron microscopy graphs for fluidMAG/C11-D.

A. Nanoparticle tracking analysis size versus concentration graph. Here, the nanoparticles were diluted to 10 µg/mL in particle-free water (Sigma Aldrich, Ireland) and analysed through 5, 60 s recordings. B. Dynamic light scattering graphs depicting size distribution and apparent zeta potential at pH 7. Zeta potential data was provided by the suppliers, Chemicell (C). For size measurements with DLS, the nanoparticles were diluted to 50 µg/mL in particle-free water. The nanoparticles are then analysed with 10 measurements of 12 runs. D. Size distribution graph of 200 individual nanoparticles imaged via transmission electron microscopy and analysed through ImageJ software.

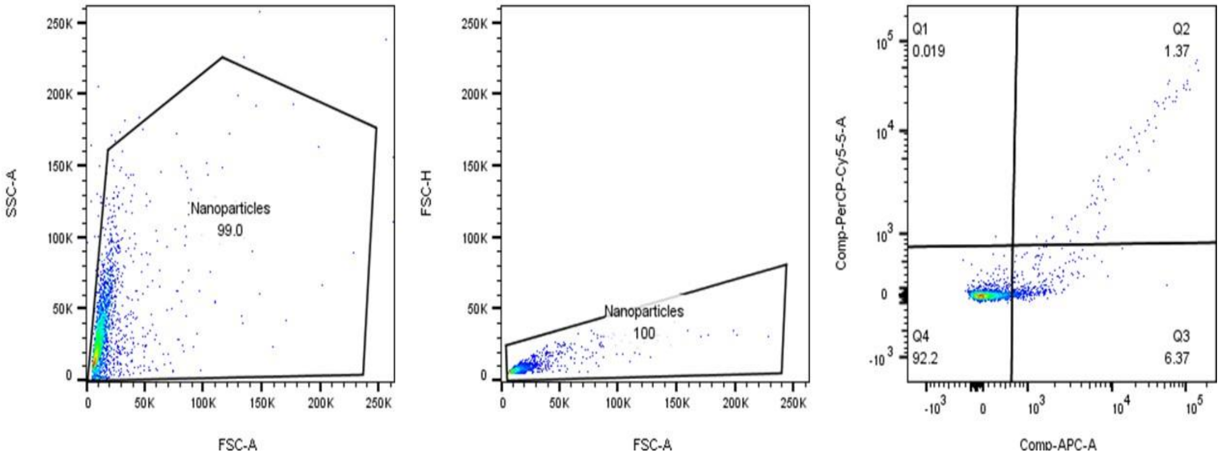

Figure S2: Nanoparticle shows little interference with APC or PerCP-Cy5.5 channels.

After gating for 99% of nanoparticles, less than 10% of the population were shown to interfere with APC and PerCP-Cy5.5 channels. It is also important to note that care was taken to avoid any nanoparticles when gating BxPC-3 cells in these experiments.

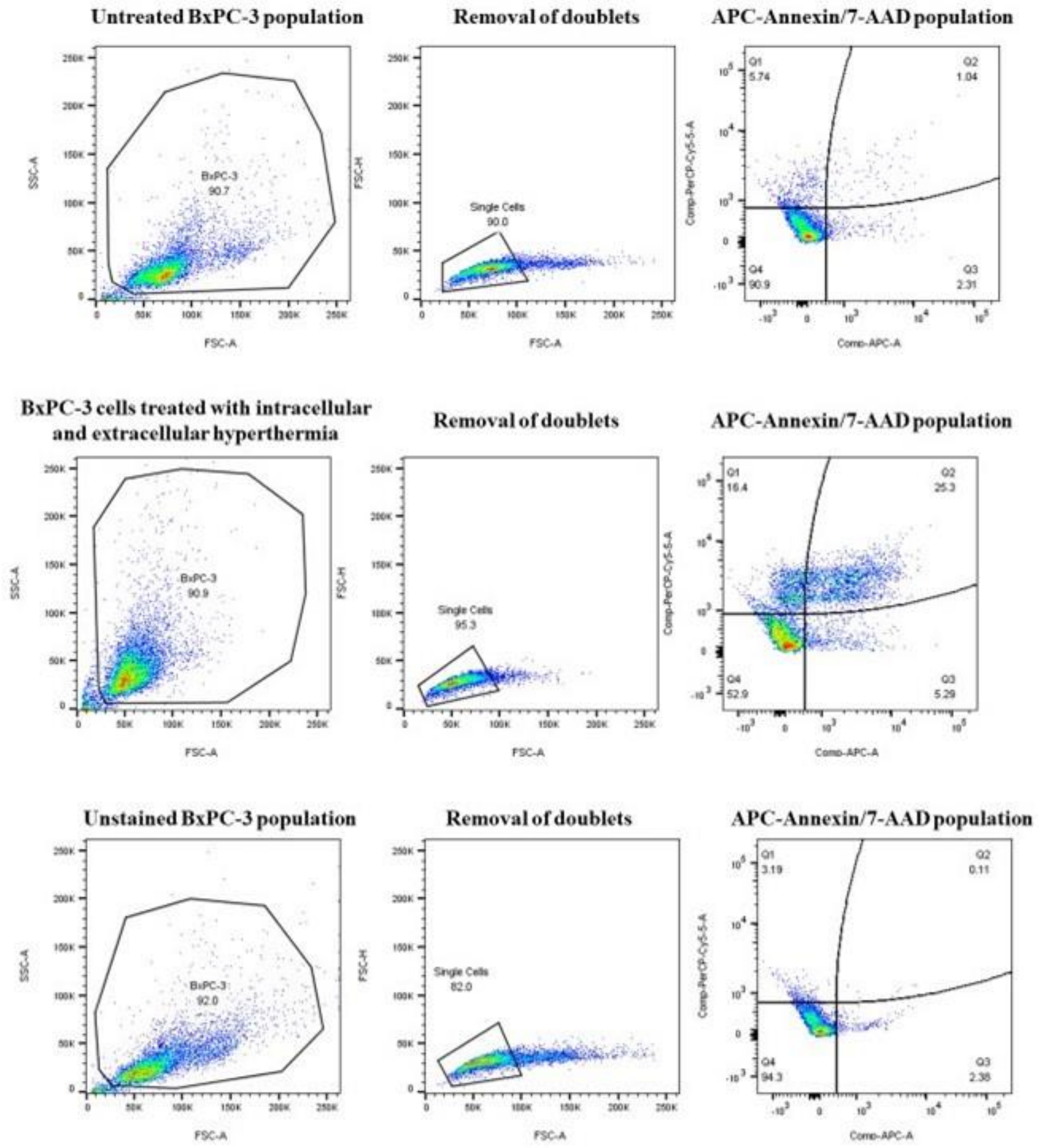

Figure S3: Gating strategy for BxPC-3 cells.

BxPC-3 treated with nanoparticles were gated to ensure nanoparticle avoidance. Doublets are removed through forward scatter height versus forward scatter area. BxPC-3 cells APC^{-}/PerCP-Cy5-5^{-}

were considered vital, APC^{+}/PerCP-Cy5-5^{-} were considered early apoptotic, APC^{+}/PerCP-Cy5-5^{+} were considered late apoptotic and APC^{-}/PerCP-Cy5-5^{+} were considered necrotic.

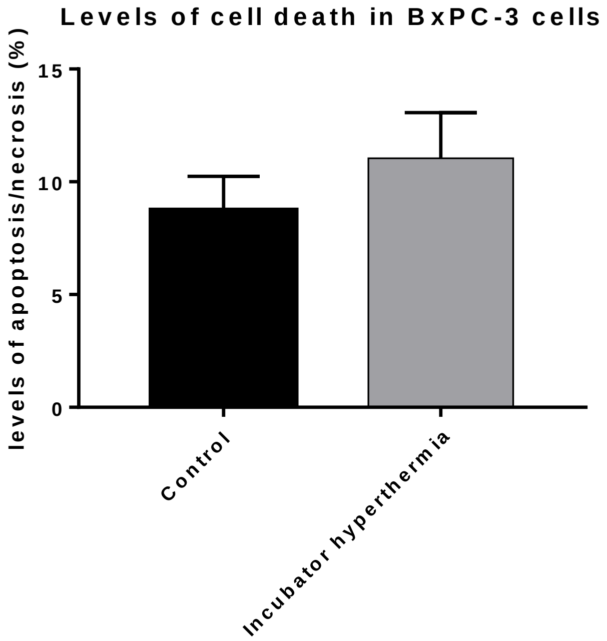

Figure S4: Annexin V/7-AAD staining of cells treated with incubator hyperthermia (42.5 °C for 30 min) versus untreated.

The levels of apoptosis and necrosis in incubator heated cells were not significantly different to untreated cells. N = 5 (triplicate). Analysed with unpaired t-test.
